# Topical Delivery of Tenofovir Disoproxil Fumarate and Emtricitabine from Pod-Intravaginal Rings Protects Macaques from Multiple SHIV Exposures

**DOI:** 10.1371/journal.pone.0157061

**Published:** 2016-06-08

**Authors:** Priya Srinivasan, John A. Moss, Manjula Gunawardana, Scott A. Churchman, Flora Yang, Chuong T. Dinh, James M. Mitchell, Jining Zhang, Rob Fanter, Christine S. Miller, Irina Butkyavichene, Janet M. McNicholl, Thomas J. Smith, Marc M. Baum, James M. Smith

**Affiliations:** 1 Laboratory Branch, Division of HIV/AIDS Prevention, Centers for Disease Control and Prevention, Atlanta, Georgia, United States of America; 2 Department of Chemistry, Oak Crest Institute of Science, Monrovia, California, United States of America; 3 Auritec Pharmaceuticals, Inc., Pasadena, California, United States of America; 4 Total Solutions, Inc., Atlanta, Georgia, United States of America; University of California, San Francisco, UNITED STATES

## Abstract

Topical preexposure prophylaxis (PrEP) against HIV has been marginally successful in recent clinical trials with low adherence rates being a primary factor for failure. Controlled, sustained release of antiretroviral (ARV) drugs may help overcome these low adherence rates if the product is protective for extended periods of time. The oral combination of tenofovir disoproxil fumarate (TDF) and emtricitabine (FTC) is currently the only FDA-approved ARV drug for HIV PrEP. A novel pod-intravaginal ring (IVR) delivering TDF and FTC at independently controlled rates was evaluated for efficacy at preventing SHIV162p3 infection in a rigorous, repeat low-dose vaginal exposure model using normally cycling female pigtailed macaques. Six macaques received pod-IVRs containing TDF (65 mg) and FTC (68 mg) every two weeks, and weekly vaginal exposures to 50 TCID_50_ of SHIV162p3 began one week after the first pod-IVR insertion. All pod-IVR-treated macaques were fully protected throughout the study (P = 0.0002, Log-rank test), whereas all control animals became infected with a median of 4 exposures to infection. The topical, sustained release of TDF and FTC from the pod-IVR maintained protective drug levels in macaques over four months of virus exposures. This novel and versatile delivery system has the capacity to deliver and maintain protective levels of multiple drugs and the protection observed here warrants clinical evaluation of this pod-IVR design.

## Introduction

Since the beginning of the AIDS epidemic, almost 78 million people have become infected with HIV. More than 50% of those living with HIV are women, with more than 350,000 new infections in young women (15–24 years old) estimated to occur each year [1]. Most of these infections were acquired through heterosexual intercourse. With an HIV vaccine still years away, the use of antiretroviral (ARV) drugs in preexposure prophylaxis (PrEP) is a promising biomedical intervention to prevent new infections. The choice of drug(s) available for HIV PrEP has expanded greatly in recent years, and many ARVs that were historically used exclusively for treatment have been added to the PrEP pipeline [2–4]. ARV drugs from multiple classes, including reverse transcriptase inhibitors (both nucleoside and nonnucleoside), entry inhibitors, integrase inhibitors, and protease inhibitors, are being evaluated in preclinical models to assess their effectiveness as PrEP agents [2, 5–9]. However, Truvada^®^, the oral formulation of tenofovir disoproxil fumarate (TDF) and emtricitabine (FTC), is the only ARV drug currently approved by the FDA for use in PrEP [10].

Clinical trials utilizing PrEP to reduce HIV transmission in recent years have demonstrated varying levels of efficacy, with the highest success observed in trials using orally administered Truvada® [11–16]. When used effectively, PrEP reduces the risk of HIV acquisition by 44–86% in studies of heterosexual women and men, men-who-have-sex-with-men and injecting drug users [11, 14, 17]. Unfortunately, in two large studies of women (VOICE and FEMPrEP), oral dosing with Truvada^®^ was not efficacious [3, 11, 18, 19]. Topical vaginal delivery of ARV drugs using gels, vaginal rings, vaginal tablets or films offer an alternative method of HIV prevention that is female-controlled. While vaginal application of tenofovir (TFV) gel reduced HIV acquisition overall by 39%, and 54% in high adherers in the CAPRISA 004 PrEP study [20, 21], this success was not replicated in two other clinical trials, VOICE and FACTS001 [18, 22, 23]. A detailed analysis of drug levels measured in samples from study participants in oral and topical dosing groups revealed a striking correlation between adherence and protection. These findings are important in two aspects. First, the trials have provided proof of concept that Truvada^®^ can be highly effective at preventing HIV infection. Second, PrEP efficacy is highly correlated with adherence, and adherence should be a primary concern when contemplating which PrEP modalities may have the most impact on the epidemic. Realistically there is no single best option; oral dosing may be preferred by some at risk populations, and topical PrEP presents several options, both coitally dependent and independent. Also on the horizon are the first trials for long-acting injectable PrEP [24, 25].

An intravaginal ring (IVR) delivering a combination of TDF and FTC in a coitally independent, sustained-release formulation leverages the success of the Truvada^®^ clinical trials and its subsequent approval for oral PrEP, with the potential for increased adherence to a monthly IVR compared to daily oral dosing. We have developed a novel pod-IVR formulation delivering a combination of TDF and FTC and evaluated the pharmacokinetics (PK) and preliminary safety of sustained delivery over 28 days in macaques [26]. The pod-IVR design allows for customized, independent release rates for multiple drugs from a single platform [26–30]. Here, we demonstrate that TDF-FTC pod-IVRs provide complete protection from SHIV162p3 infection over 16 weeks in a rigorous repeat low-dose challenge model in pigtailed macaques.

## Materials and Methods

### Ethics Statement

This research was approved by the CDC Institutional Animal Care and Use Committee (Protocols 2444SMIMONC and 2445SMIMONC). All animals were housed in an AAALAC accredited (AAALAC #00052 and PHS assurance #A4365-01) facility with husbandry and enrichment under the direction of attending veterinarians (AV) and a nonhuman primate enrichment coordinator within the CDC Animal Resources Branch (ARB) using established standard operating procedures. All macaques are monitored several times daily by ARB staff and undergo physical examinations twice per year. Fecal samples are routinely tested for enteric pathogens and animals that are found to be lethargic or exhibit other signs of illness are examined and treated at the discretion of the AV. Cage sizes (dimensions 30 inches height x 30 inches width x 30 inches length) are as dictated by the Animal Welfare Act and as outlined in the Animal Resources Branch Environmental Enhancement plan. Macaques are pair housed when possible by species and gender, and under protected contact housing when necessary for the study. Standard enrichment includes: Objects to manipulate in cage, including durable objects and paper, cage complexities such as perches or swings, varied food supplements such as fruits, vegetables, or seeds, foraging or task-oriented feeding method, and human interaction by caregiver and research staff. No macaques were sacrificed during or after this study. All macaques were transferred to animal care and use protocols within CDC for further pharmacokinetic studies with different PrEP products. In cases where the animal is displaying signs of discomfort, but has not reached the threshold for euthanasia as determined by the AV, pain relief in the form of meloxicam, 0.2 mg SQ on day one followed by 0.1 mg/kg PO thereafter for 5 days or as needed, is generally used. Secondary infections (e.g., diarrhea) are treated as required at the discretion of the AV. Behavioral abnormalities are also treated at the discretion of the AV. The PI is consulted if the condition cannot be managed medically and if so, animals are euthanized to prevent further trauma in accordance with the 2000 Report of the AVMA Panel on Euthanasia (http://www.avma.org/issues/animal_welfare/euthanasia.pdf).

### Materials

Tenofovir disoproxil fumarate (TDF) and emtricitabine (FTC) were kindly provided by Gilead Sciences, Inc. (Foster City, CA), under a material transfer agreement (MTA) dated August 8, 2011. Tenofovir, [adenine-^13^C_5_] (TFV-^13^C_5_) was obtained from Moravek Biochemicals, Inc. (Brea, CA) and maraviroc-D6 (MVC-D_6_) was obtained from Santa Cruz Biotechnology, Inc. (Dallas, TX).

### Manufacture of pod-IVRs

Macaque sized pod-IVRs for this study were produced using identical methods to those described previously [26]. Briefly, the IVRs contained 3 pods each of TDF (64.8 ± 2.2 mg) and FTC (67.6 ± 1.3 mg) inserted into a polydimethylsiloxane (silicone) blank ring with mechanically fashioned delivery channels as specified in Table 1.

**Table 1:** Characteristics of TDF-FTC pod-IVRs used in pig-tailed macaque studies.

	TDF	FTC
Drug loading (mean +/- SD) (mg)	64.8 ± 2.2	67.6 ± 1.3
Number of pods/ring	3	3
Delivery channels/pod	1	1
Delivery channel cross-sectional area (mm^2^)	1.77	0.79
*In vitro* release rate (mean ± SD) (mg day^-1^)^a^	1.2 ± 0.25	9.2 ± 0.44
*In vivo* release rate (mean ± SD) (mg day^-1^)^b^	0.73 ± 0.13	2.87 ± 0.41

^a^ n = 6

^b^ n = 54, calculated from residual drug in pod IVRs from efficacy study

### Measurement of *in vivo* TDF-FTC release

The *in vivo* release of TDF and FTC was determined from the amount of residual drug in used IVRs. Silicone segments containing one pod each were cut from the used IVRs. Each segment was cut open to expose the remaining pod residue, placed in 100 mL of 50% v/v aqueous methanol, and stirred overnight to dissolve the remaining drug in the pod. The concentration of TDF, TI (tenofovir isoproxil), TFV, and FTC in each solution was measured by high-performance liquid chromatography (HPLC) with UV detection (1100 Series, Agilent Technologies). Sample liquid chromatography-tandem mass spectrometry (LC-MS/MS) chromatogram overlays from a vaginal fluid sample collected during the TDF-FTC pod-IVR efficacy trial are shown (S1 Fig). A Waters (Milford, MA) Atlantis T3 C18 column (2.1 × 100 mm; 5 μm) controlled at 30°C was used as the stationary phase. The following gradient program was used (A, 1.0% v/v acetic acid and 3.0% v/v acetonitrile in water; B, acetonitrile): 1 min 100% A; 2 min ramp from 100:0 A:B to 75:25 A:B; 3 min hold at 75:25 A:B; 2 min ramp from 75:25 A:B to 100:0 A:B; 2 min hold at 100% A. The detection wavelength was 260 nm for TDF, TI, and TFV and 280 nm for FTC. Retention times were 8.29 min (TDF), 5.24 min (TI), 3.37 min (FTC), and 1.12 min (TFV). The method run times were 10 min.

### Quantification of TDF, TFV, and FTC vaginal fluid concentrations

Sample purification and analysis was carried out at Oak Crest using the following validated protocols. Weck Cels containing a known mass of absorbed, undiluted vaginal fluid were extracted with aqueous methanol (50% v/v, 1 ml) with vortex agitation for 5 min, sonication for 5 min, vortex agitation for 5 min, and centrifugation at 12,000 *g* for 10 min. An aliquot (0.75 ml) of the supernatant was transferred into a 96-well plate, evaporated to dryness in a SpeedVac concentrator system (Savant SC210A Plus, Thermo Fisher Scientific, Inc., Hudson, NH), and reconstituted with 0.1% v/v formic acid in water (500 μl). Aliquots (100 μl) of the reconstituted extract were dispensed into 96-well plates for TFV analysis while another set of aliquots (200 μl) was dispensed into separate 96-well plates for TDF and FTC analysis. Each set of samples also included a minimum of six standards and a minimum of three quality controls in accordance with FDA guidelines [31]. Samples were spiked with internal standard (IS) solution (MVC-D_6_ for TDF-FTC and TFV-^13^C_5_ for TFV) at a final concentration of 1 μg ml^-1^.

For TDF-FTC, sample purification was carried out in 96-well format using a reversed phase C-18 solid phase extraction system (Strata-X, Phenomenex, Inc., Torrance, CA) according to the manufacturer’s instructions. For TFV, sample purification was carried out in 96-well format using a mixed-mode anion exchange and reversed-phase copolymeric sorbent system (Oasis MAX, Waters Corporation, Milford, MA) according to the manufacturer’s instructions. The purified samples were dried *in vacuo* using the SpeedVac concentrator described above and were reconstituted in 0.1% v/v formic acid in water (200 μl for TDF-FTC; 100 μl for TFV) prior to analysis.

The concentration of TDF and FTC was measured by LC-MS/MS using an HPLC system consisting of a model G1367A well-plate autosampler, G1312A binary pump, and G1316 thermostatted column compartment (1200 Series, Agilent Technologies, Santa Clara, CA) interfaced to an API 3000 triple quadrupole mass spectrometer (AB Sciex, Framingham, MA) with a Turbo Ion Spray electrospray ionization source. An Agilent Zorbax Eclipse XDB-C18 Rapid Resolution column (2.1 × 50 mm; 3.5 μm) controlled at 40°C was the stationary phase. The following gradient program was used (A, 0.1% v/v formic acid in water; B, 0.1% v/v formic acid in acetonitrile): 0.25 min 100% A; 0.25 min ramp from 100:0 A:B to 95:5 A:B; 1.5 min ramp from 95:5 A:B to 70:30 A:B; 1.5 min hold at 70:30 A:B; 1.5 min ramp from 70:30 A:B to 95:5 A:B; 0.5 min ramp from 95:5 A:B to 100:0 A:B resulting in a total run time of 5.5 min. Retention times were 3.80 min (TDF) and 0.25 min (FTC). The following ion transitions were measured using electrospray ionization (ESI)+ ionization (*m/z*, parent → product) TDF, 520.0 → 270.0; FTC, 248.0 → 130.0; MVC-D_6_ (IS), parent 520.7 → 280.6 (S1 Fig). Due to the high concentration of FTC in the samples, these were diluted 100-fold and reanalyzed.

The concentration of TFV was measured by LC-MS/MS using the above instrumentation and stationary phase. The following gradient program was used (A, 0.1% v/v formic acid in water; B, 0.1% v/v formic acid in acetonitrile): 0.25 min ramp from 100:0 A:B to 95:5 A:B; 0.5 min ramp from 95:5 A:B to 100:0 A:B; 0.25 min hold at 100:0 A:B resulting in a total run time of 1.0 min, with a TFV retention time of 0.2 min. The following ion transitions were measured using ESI+ ionization (*m/z*, parent → product): TFV, 288.1 → 176.2; TFV-^13^C_5_ (IS), 293.1 → 181.2.

Both methods used standard curves consisting of seven or more points (1–7,500 ng ml^-1^ TDF, 1–10,000 ng ml^-1^ TFV, 1–750 ng ml^-1^ FTC) prepared in vaginal fluid diluted in 50% v/v aqueous methanol and processed as above. All standard curves were linear with *R*^*2*^ values greater than 0.99. The lower limits of quantification (LLQ) in the analyzed Weck Cel extracts were: TDF; 1 ng ml^-1^; TFV, 5 ng ml^-1^; FTC, 5 ng ml^-1^. Three separately prepared quality control samples were analyzed at the beginning and end of each sample set to ensure accuracy and precision within 20%, in accordance with FDA bioanalytical validation criteria [31].

### In vitro release studies

*In vitro* release of TDF into a simplified vaginal fluid simulant (VFS) was carried out on IVRs identical to those used previously [26]. The VFS was adapted from Owen and Katz and consisted of 25 mM acetate buffer (pH 4.2) with NaCl added to yield a 200 mOs solution [32]. For all *in vitro* release studies, the IVRs were placed in glass jars containing 100 mL VFS at 37 ± 0.5°C with shaking at 60 rpm on an orbital shaking incubator. Aliquots (100 uL) of release medium were removed daily, and the release medium replaced completely with 100 mL fresh VFS following the day 7 sampling.

The concentration of TDF and FTC in each daily aliquot was determined by HPLC using the same method as for *in vivo* TDF and FTC release measurement described above. For all *in vitro* analyses, we quantify by HPLC-UV the parent TDF and the hydrolysis products TI and TFV that are formed upon hydrolytic loss of one and two isoproxil groups, respectively. Typically, < 5% of the TDF in the release media after 28 days is hydrolyzed to TI, and TFV is not observed.

### Macaque study

Efficacy studies were conducted as previously described at the Centers for Disease Control and Prevention (CDC) under approved CDC Institutional Animal Care and Use Committee and standard guidelines according to the Guide for the Care and Use of Laboratory Animals (National Research Council of the National Academies, 2010). Briefly, six sexually mature and normally cycling female pigtailed macaques had pod-IVRs containing TDF (65 mg total, 3 pods) and FTC (68 mg total, 3 pods) placed in the vaginal vault close to the cervix 7 days prior to the first vaginal viral exposure to SHIV162p3. The delay in virus exposure was to ensure that the macaques were used to the rings and did not remove them. The rings were exchanged every 2 weeks (3 days prior to the next weekly virus exposure) corresponding to a total of eight ring changes during the 18 week period, with the final ring removal at week 18. The group of six macaques with pod-IVRs along with nine normally cycling pigtailed macaque controls (3 real-time and 6 historical) were exposed to weekly atraumatic vaginal inoculations of 50 TCID_50_ SHIV162p3 for a maximum of 16 weeks or until viral infection was confirmed on two consecutive weeks with real-time PCR detection in plasma (limit of detection 50 copies viral RNA/mL of plasma) [33]. The challenge virus (SHIVSF162p3) stock, obtained from the NIH AIDS Reagent Program, was propagated in con A stimulated- pigtailed macaque peripheral blood mononuclear cells (PBMC) and titered on pigtailed macaque PBMC. Ketamine was routinely used to anaesthetize the animals (3-10mg/kg). The anaesthetized macaques were maintained in a recumbent position for at least 15 minutes after vaginal viral inoculations. All infections were confirmed by serology (Genetic Systems HIV-1/HIV-2, Bio-Rad, and SIV Western Blot Assay, Zeptometrix). Vaginal fluids were obtained with Weck-Cel spears proximal and distal to the ring location at every ring exchange for quantitation of TFV and FTC by LC-MS/MS described here and according to published protocols [26, 34]. The menstrual cycle of the pigtailed macaques was monitored with weekly plasma progesterone measurements. The plasma progesterone analyses were performed by the University of Wisconsin National Primate Research Center.

### Statistical Analysis

Data were analyzed using GraphPad Prism (version 6.02; GraphPad Software, Inc., La Jolla, CA). *In vivo* TDF and FTC release rates for all pod-IVRs used in the study were compared using an unpaired Student t-test with Welch’s correction. Statistical significance was defined as a P value of ≤0.05.

## Results

### Pod-IVR fabrication and in vitro release kinetics

**Fig 1:**
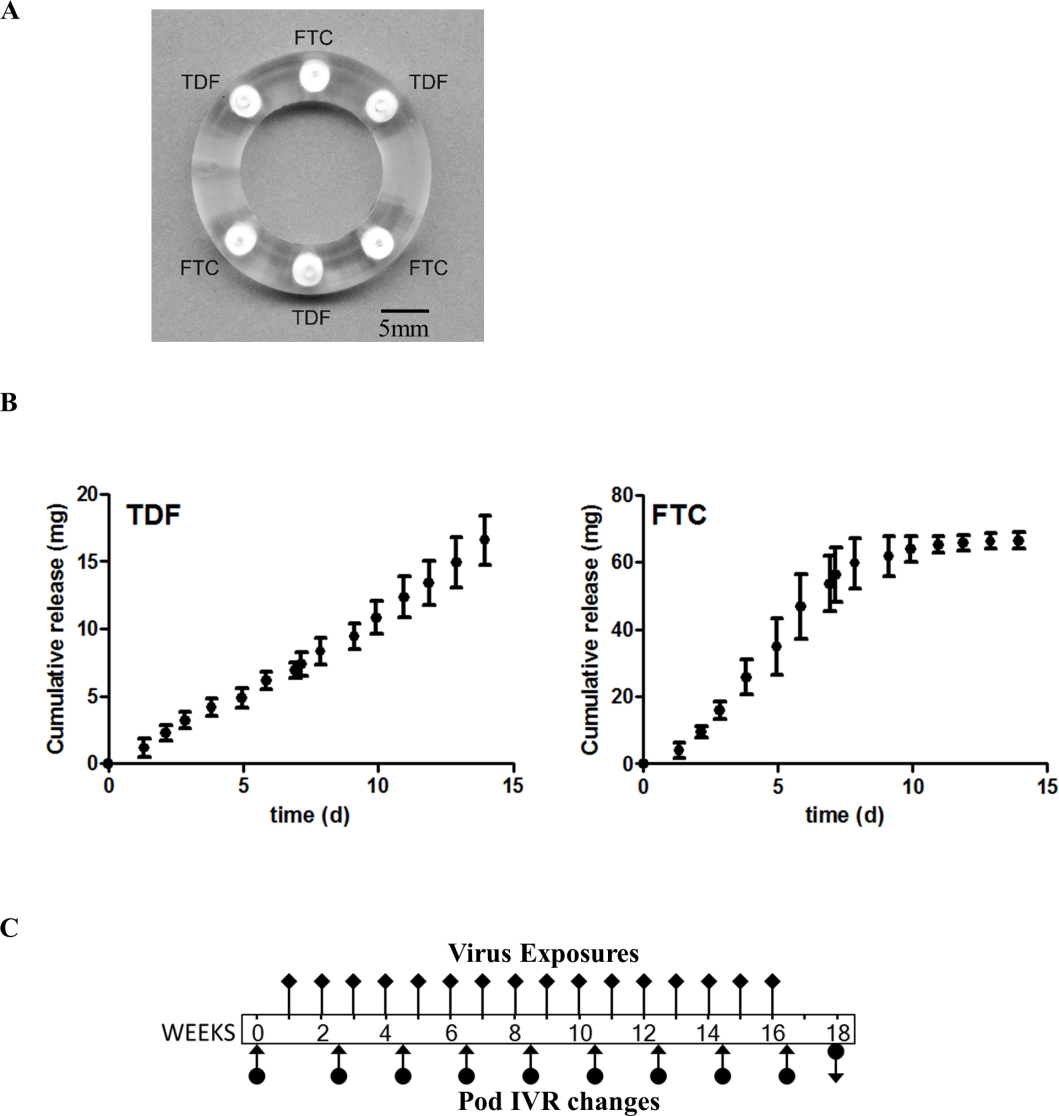
**Macaque pod-IVR, in vitro release, and study design** (A) Macaque TDF-FTC pod-IVR with three pods each of TDF and FTC. (B) Cumulative *in vitro* release over 14 days shows near linear release of TDF and FTC from the pod IVR. (C) Macaque efficacy study timeline. Pod-IVRs were inserted one week prior to the first vaginal virus exposure and were changed every two weeks (arrows with filled circles), 3 days prior to the next virus exposure. The last pod-IVR was removed (indicated by a downward arrow) at week 18. Vaginal fluids were collected at each ring change for pharmacokinetic evaluation. Atraumatic vaginal exposures to 50 TCID50 of SHIV162p3 were administered once weekly (black diamonds) and blood was collected prior to each exposure to monitor for plasma viral loads.

Pod-IVRs containing three TDF and three FTC pods were identical to those used in a comprehensive PK and preliminary safety study [26] and were fabricated using methods described in detail previously [26, 27]. The macaque pod-IVR dimensions (Fig 1A) were based on the optimal design determined in a previous size-fitting experiment in pigtailed and rhesus macaques [35]. The number of drug pods and delivery channel configuration of each pod were chosen to target an *in vivo* release rate of 0.7 mg/day TDF and 2.5 mg/day FTC [26]. *In vitro* release profiles for TDF and FTC exhibited zero order release kinetics (Fig 1B), in accordance with a mechanistic analysis of pod-IVR drug release [36]. Note that to achieve the target FTC *in vivo* release rate, the corresponding *in vitro* release rate was significantly higher (Fig 1B, Table 1) and depleted most of the drug reservoir within 10 days. This was not the case *in vivo*, where on average 43.5% of the FTC drug loading (30.4 ± 12.9 mg, mean ± SD) remained following IVR removal after 14 days. The in vivo release rates are consistently lower than the in vitro release rates, and this has been observed in sheep, macaques, and humans for TDF and FTC. The in vitro release rate is merely a quality control, not a predictor of in vivo release rates.

### Pharmacokinetics of TDF and FTC delivery from pod-IVRs over 4 months

**Fig 2:**
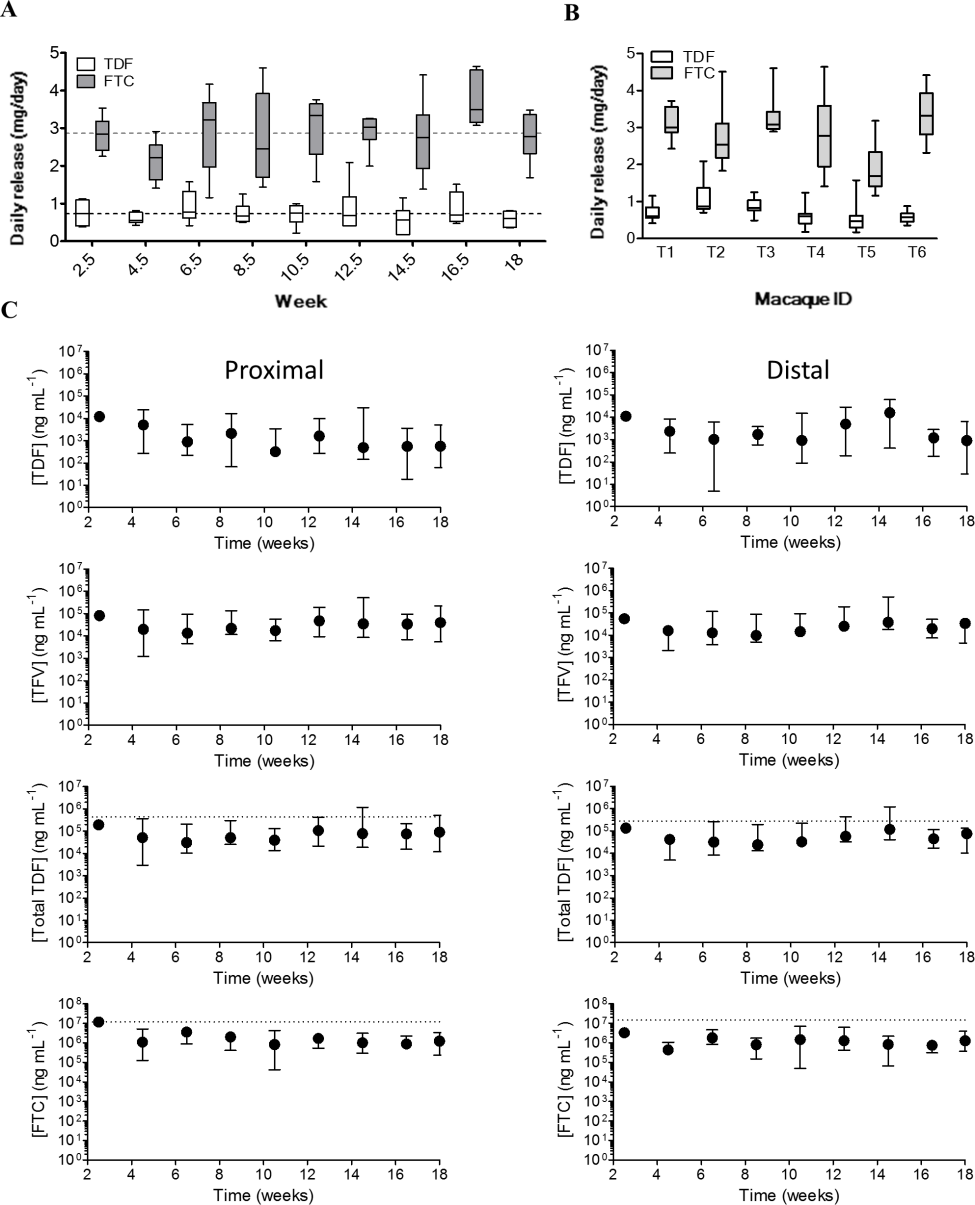
**Pod-IVR Pharmacokinetics in macaques** (A) *In vivo* release of TDF and FTC from each pod-IVR (N = 6/time point) over the course of the efficacy study determined by residual drug measurements from the pod-IVRs that were in place for 19 weeks with IVRs exchanged for new devices every 2 weeks. The top and bottom of the boxes show the 75th and 25th percentiles, respectively, and the line in the middle of the box is the median value. The dotted lines show the mean (N = 6) *in vivo* release from identical pod-IVRs obtained during the PK study preceding this efficacy study [26]. (B) *In vivo* release profile for individual macaques (T1-T6) shows variability between animals. (C) TDF, TFV, TDF+TFV, and FTC levels in vaginal fluids collected at each ring exchange. Vaginal fluids were collected with Weck-Cel sponges proximal and distal to the pod-IVR placement. The dotted horizontal lines correspond to the medians from our previous PK study [26]. Left panels-proximal; Right panels-distal; Dots-median.

The study timeline is illustrated in Fig 1C. Pod-IVRs were inserted at the beginning of the 18-week study period, with IVRs exchanged for new devices every 2 weeks, 3 days following virus exposure. Daily TDF and FTC release rates were calculated from the amount of residual drug in each pod-IVR following removal, and assuming linear release over the two week insertion period (Fig 2A). Quantitation of residual drug from all 54 pod-IVRs collected from the macaques over the 18 week efficacy study afforded a mean *in vivo* release of 0.73 ± 0.13 mg/day for TDF and 2.87 ± 0.41 mg/day for FTC (Table 1). The residual drug in the pod-IVRs after removal consisted of 93.5% TDF, 4% TI, and 2.5% TFV (calculated on a molar basis, average for N = 54 pod-IVRs). A range of *in vivo* release rates was observed over the course of the study (Fig 2A), but there was no statistically significant difference between the release from pod-IVRs in the efficacy study compared to the earlier PK study using the same formulation (TDF: P = 0.670, FTC: P = 0.209) as determined using an unpaired Student t test with Welch’s correction [26]. The variation observed when the macaques were analyzed as a group (Fig 2A) can partially be explained in terms of physiological variability between animals (Fig 2B), as discussed elsewhere [26].

The concentration of TDF, TFV and FTC in vaginal fluid was measured at each ring exchange. Vaginal fluid drug levels were above the LLQ of the assay in 100% of the samples for TDF (98/98), 98% of the samples for TFV (96/98), and 97% of the samples for FTC (95/98). Four samples contained 5 mg of vaginal fluids or less and were omitted from the analysis. The measured levels exhibited the expected variability (Fig 2C), with FTC concentration (median: 1100 μg/mL; interquartile range (IQR): 530–2,400 μg/mL) consistently several log units higher than the drug’s EC_50_ (0.0003–0.16 μg/mL)[37], and TDF/TFV concentration (median: 46 μg/mL, IQR: 27–137 μg/mL) consistently above the 1,000 ng/mL TFV concentration in cervicovaginal fluid samples that correlated with protection from HIV infection in the CAPRISA 004 trial [38]. Due to the expected hydrolysis of TDF to TFV in vaginal fluids following release from the pod-IVR, TDF concentrations in vaginal fluids were approximately 36 times lower than corresponding TFV concentrations (Fig 2C).

### Complete protection from vaginal SHIV infection

**Fig 3:**
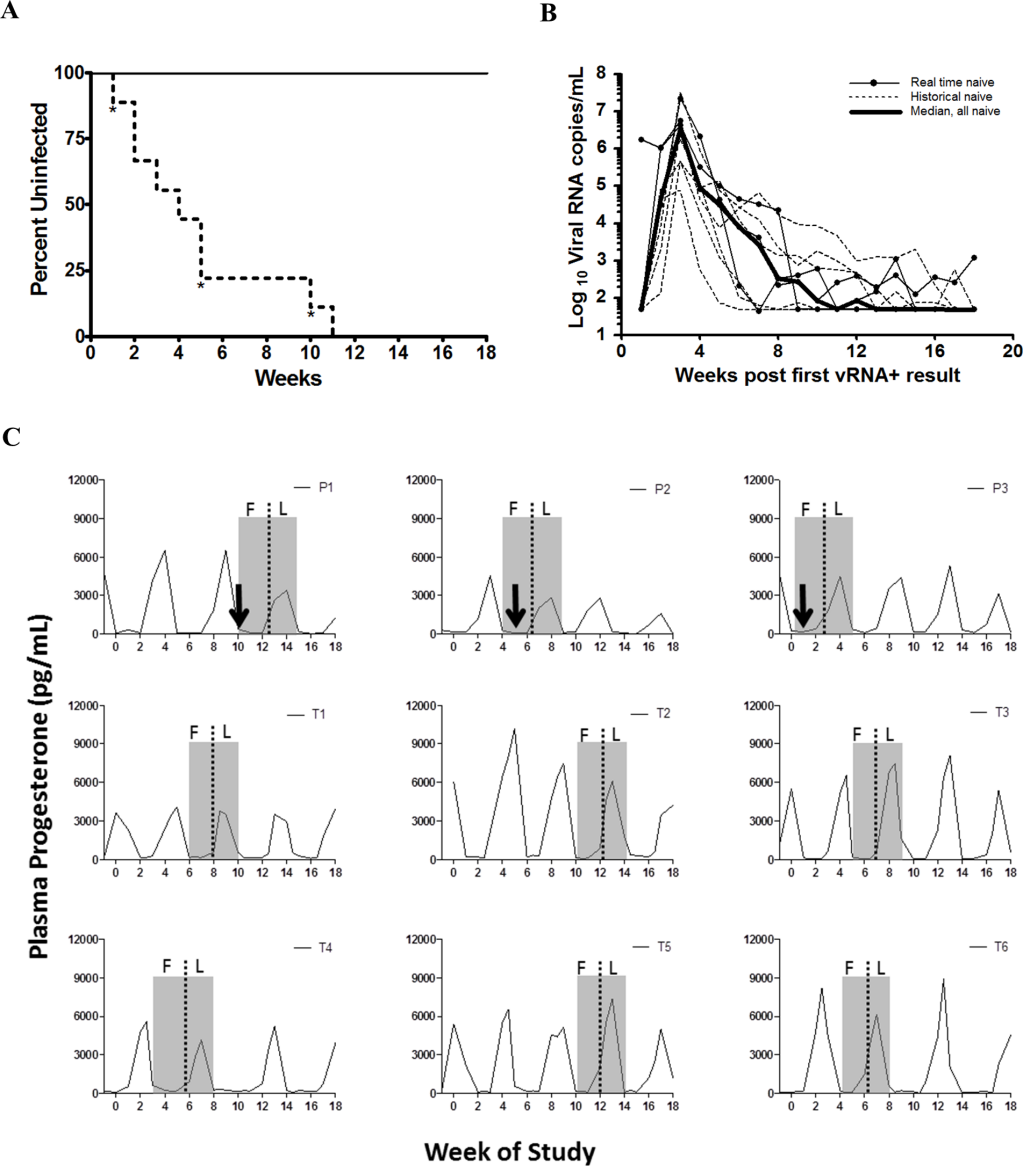
**TDF-FTC pod-IVR efficacy in macaques** (A) Kaplan-Meier plot showing complete protection for 18 weeks in TDF-FTC group (n = 6, solid line) compared to naïve group (n = 6 historical controls, n = 3 real time controls, dotted line. * indicates time of infection for real time controls). P = 0.0002, Log rank test. (B) Peak viral loads of infected controls (dotted lines, historical controls; narrow solid lines, real time controls; bold line, median. Limit of detection was 50 viral RNA copies/mL of plasma. (C) Relationship of menstrual cycle and time of infection. Macaque plasma was monitored for progesterone over the course of the challenge study to confirm menstrual cycles and determine timing of infection. Day 1 of the menstrual cycle corresponds to the steep drop in progesterone after peak level for that month (shaded boxes represent one cycle: F—follicular, L—luteal). Arrows depict time of infection assuming a one week eclipse period from infection to detection of viral RNA in plasma. P1-P3, real-time naïve control animals; T1-T6, pod-IVR-treated animals.

The ability of TDF-FTC pod-IVRs to prevent infection by SHIV162p3 was evaluated in a repeat, low-dose challenge model. Six macaques treated with TDF-FTC pod-IVRs received weekly atraumatic vaginal SHIV162p3 virus exposures of 50 TCID_50_ for 16 weeks beginning one week after insertion of the first pod-IVR (Fig 1C). All six treated macaques remained negative for plasma viral RNA and seronegative throughout the 18 week study period (Fig 3A, S1 Table). All treated animals remained seronegative and also negative for plasma viral RNA 18 months after the first exposure. In contrast, the three real-time, naïve controls undergoing identical viral challenge were infected at rate similar to the six historical controls; median of 5 exposures to infection versus 4 with historical controls, and 4 when the two control groups were combined (p = 0.7154, Unpaired Student t test with Welch’s correction) [39]. Median peak viral load of infected animals was 3.40 x 10^6^ viral RNA copies/mL of plasma, and the subsequent decline in viral load was similar to that observed in cycling pigtailed macaques when using this viral stock (Fig 3B) [40]. The window of highest susceptibility to SHIV infection in cycling pigtailed macaques has been reported to occur in the late luteal/early follicular phase [41, 42]. All macaques in this study received virus exposures over at least 3 full menstrual cycles and the controls were all infected during the early follicular phase (Fig 3C).

## Discussion

This study demonstrates for the first time that TDF in combination with FTC can be fully protective when delivered topically by a sustained release IVR device. Using the well-characterized and rigorous pigtailed macaque repeat low-dose challenge model, protection was achieved in 6 of 6 animals with TDF-FTC pod-IVRs compared to 0 of 9 control animals without pod-IVRs over four months of weekly SHIV162p3 virus exposures that spanned several menstrual cycles. The observed protection in macaques indicates the significant potential for the combination of TDF and FTC delivered from a pod-IVR to prevent sexual HIV infection.

The selection of ARV drugs for delivery from IVRs needs to take into account physiochemical properties of the compounds, including compatibility with the IVR elastomer, and pharmacologic properties that affect distribution, uptake, and clearance in the relevant anatomic compartments (vaginal fluid, mucosal tissue, local CD4^+^ immune cells, and plasma) and the mode of action of the drug against HIV. The TDF-FTC combination was chosen for a number of reasons. Truvada^®^, a once-daily oral combination of TDF and FTC, is the only FDA-approved ARV drug regimen for HIV PrEP, significantly reducing the regulatory burden of approval for a topical TDF-FTC combination compared to ARV agents such as TFV, MIV-150 and MIV-160, or dapivirine that are not approved for oral or topical use in either HIV prevention or treatment. The oral TDF-FTC combination has demonstrated success in PrEP clinical trials. Although the systemic and mucosal PK are much different for oral versus topical dosing with the TDF-FTC combination the mucosal levels achieved with this IVR far exceed what is seen in macaques with oral dosing [39]. Pharmacologically, both TDF and FTC are nucleos(t)ide analogue reverse transcriptase inhibitors (NRTIs), and their active forms are their diphosphate and triphosphate anabolites TFV-DP and FTC-TP, which act intracellularly with long half-lives of 139–150 h and 39 h, respectively, in humans, and estimated to be 58h and 29h in pigtailed macaques [39, 43, 44]. The long intracellular half-lives can result in protective levels being maintained even with poor adherence and intermittent dosing [45, 46]. Additionally, TDF is the disoproxil diester prodrug of TFV and exhibits significantly greater tissue uptake from vaginal fluids than the parent TFV, although TFV delivered topically can also be protective as was shown in previous macaque studies with gel formulations [28, 45, 47]. The success of the TDF-FTC combination in HIV PrEP can be leveraged further by the addition of a third ARV drug from a different mechanistic class [43, 48, 49]. The additive, and potentially synergistic, effect of a triple combination IVR is readily achieved by the versatile nature of the pod-IVR design [26, 27, 30, 50]. The addition of a third ARV may become necessary in the future if resistance to TDF-FTC becomes a public health issue due to intermittent use of Truvada^®^ in prevention and treatment[51].

The novel pod-IVR platform embodies several important advantages for the controlled, sustained, topical delivery of ARV agents. The pod-IVR design overcomes the limitations inherent to reservoir or matrix IVR designs by enabling customizable, individually controlled release rates from each pod independent of the elastomer material [8], as was demonstrated for a pod-IVR delivering five different drugs with widely varying physicochemical properties [30]. Pod-IVRs exhibit zero-order, independently controlled *in vitro* release kinetics for the release of >80% of the total drug load [8, 27, 50], unlike the reservoir or matrix IVR designs that exhibit significant variability in daily release rates over time.

The success of IVR-based topical PrEP for HIV prevention will depend on the ability to manufacture devices in large quantities at a cost appropriate for the developing world. The modular design of the pod-IVR facilitates a flexible, low-cost three-step manufacturing approach suitable for rapid prototyping and allowing cost-effective scale-up of manufacturing for clinical trials and eventual product production [26, 27, 34, 50]. (1) Empty silicone IVR scaffolds containing cavities for up to ten pods are fabricated by injection molding (IM) from liquid silicone resin (LSR). Delivery channels are either created during the IM process, or mechanically punched following IM prior to pod placement. (2) Drug cores are manufactured using standard pharmaceutical tableting machinery, and cores are subsequently coated with the polyvinyl alcohol (PVA) or other sustained release polymer by pan or fluidized-bed spray coating to produce pods. (3) Drug pods are placed into the pre-formed cavities in the IVR scaffolds and sealed in place with a backfill of room temperature cure silicone adhesive.

Because of its hydrolytic instability, the formulation of TDF for topical vaginal delivery is not trivial, and is challenging in aqueous gel dosage forms. Smith et al., demonstrated maintenance of protective levels of TFV in macaques when eluted from a TDF hydrophilic polyether urethane reservoir ring design with the use of an osmotic agent, but stability of the TDF in this IVR was not reported [40]. The pod-IVR protects the TDF in the solid drug core from hydrolysis to TFV. Long-term stability studies (2 years, 40°C, 70% RH) on TDF pod-IVRs showed no significant drug decomposition (<96%) during manufacture and storage [50]. The residual drug in the IVRs used in this study contained less than 6.5% of the TDF hydrolysis products TI and TFV, indicating that >93% of the released drug was in the prodrug TDF form and had not hydrolyzed prior to release from the pod-IVR. The hydrolytic stability of TDF in the pod-IVR has important implications for PK and resulting efficacy pharmacodynamics (PD) outcomes as IVR delivery of TDF has been shown to lead to approximately 100× greater tissue TFV levels compared to delivery of TFV at the same vaginal fluid concentration [28].

The results presented here have implications for products entering trials for topical HIV prophylaxis. We have shown that complete protection over multiple weeks of virus exposures can be achieved with the TDF-FTC pod-IVR in normally cycling pigtailed macaques. The TDF/TFV vaginal fluid levels maintained during the virus exposure period were more than 10× higher than the level of TFV in vaginal fluids (1,000 ng/mL) found to be protective in the CAPRISA 004 trial [38]. They were also similar to the protective levels obtained with a reservoir TDF IVR in the same macaque challenge model [40]. Ideally, the best measures for a correlate of protection are the intracellular TFV-diphosphate and FTC-triphosphate levels in mucosal tissue-resident target cells [45]. Unfortunately, these studies in macaques require multiple biopsy samples or ARV dosing coupled to necropsy, neither of which were feasible in the current study [40, 45]. When the potentially synergistic effect of the added FTC [47, 52] is taken into account, the combined vaginal fluid levels are more than sufficient to offer protection in the macaque model and should translate into similar, and hopefully protective, mucosal drug levels in women.

Female-controlled, topical HIV PrEP holds significant appeal [5, 6, 53, 54], and several clinical trials have been carried out in the hope that the success of oral PrEP could be duplicated with on demand strategies such as vaginal gels [3, 11]. It has become increasingly clear that adherence to dosing in PrEP could represent the greatest hurdle to overcome in developing an effective prevention modality [2, 55]. Adding IVRs, vaginal films, and tablets to the PrEP product development pipeline will offer women a broader range of options, which should, in turn, translate into higher adherence rates.

The repeat low-dose macaque model has been used extensively for evaluating PrEP strategies using both rectal and vaginal virus exposures in rhesus and pigtailed macaques [39, 40, 45, 46, 56–59]. The vaginal model using pigtailed macaques is best suited for IVR studies because of their larger size compared to rhesus macaques and their monthly menstrual cycles being similar to those of women. The model may also be expanded beyond the efficacy study design presented here by adding hormonal contraceptives or drugs to treat other potential risk factors such as sexually transmitted infections[59–61]. The flexibility of the pod-IVR platform and the expansive capabilities of the pigtailed macaque model will allow the investigation under controlled conditions of a variety of scenarios that may arise during clinical trials, including increased risk factors and intermittent or low adherence to PrEP products.

There are, however, limitations to this study that are inherent in all macaque efficacy studies. The virus exposures were atraumatic, and the virus dose that was administered was cell-free and without semen, unlike heterosexual HIV acquisition in women where the inoculum can be cell-associated in the presence of semen and be accompanied by micro-abrasions of vaginal tissue from sex. Based on our macaque PK study using the same IVR configuration, we observed an in vivo FTC release of 2.50 ± 0.58 mg/d. With a total FTC loading of ~68 mg and a maximum in vivo release rate of ~3.2 mg/d, and assuming that linear kinetics are observed until ~75% of the drug reservoir has been depleted, we estimated conservatively that the rings should be used for a maximum of 16 days before an exchange was necessary, instead of the proposed 30-day pod-IVR for human studies. This allowed synchronization of ring exchanges with viral challenges to ensure that drug depletion from the IVRs, especially for FTC (Fig 1C), was not greater than 75%. However, the IVRs released slower *in vivo* than *in vitro* (Fig 2A) and could have been used for 30 days. The human-sized pod-IVRs have a much larger drug loading capacity per pod, and up to 10 pods can be incorporated into a single device, providing a total drug load as large as 2 g [26].

The first efficacy study of sustained release vaginal delivery of TDF in combination with FTC, the only drugs FDA-approved for HIV PrEP, in the gold standard preclinical model demonstrated complete protection from productive SHIV infection. These results support the significant translational potential of the TDF-FTC pod-IVR as a non-vaccine biomedical prevention strategy for female-controlled HIV prevention in resource-poor regions. The TDF-FTC pod-IVR described here is currently being evaluated in an exploratory, pre-phase 1 clinical trial [62].

## Supporting Information

S1 Fig **Sample LC-MS/MS chromatogram overlays** (A) TFV (m/z, 288.1 → 176.2) and (B) TDF (m/z, 520.0 → 270.0), FTC (m/z, 248.0 → 130.0)—from a vaginal fluid sample collected during the TDF-FTC pod-IVR efficacy trial. The chromatograms were offset to illustrate the quality of the baseline.
(TIF)

S1 Table Seroconversion (EIA and western blot) of TDF/FTC and control macaques during the challenge phase. (DOCX)
