# Supplementary figures and images for: Topical Delivery of Tenofovir Disoproxil Fumarate and Emtricitabine from Pod-Intravaginal Rings Protects Macaques from Multiple SHIV Exposures
Source: PLoS One. 2016 Jun 8;11(6):e0157061. doi: 10.1371/journal.pone.0157061 (PMC4898685; doi:10.1371/journal.pone.0157061)

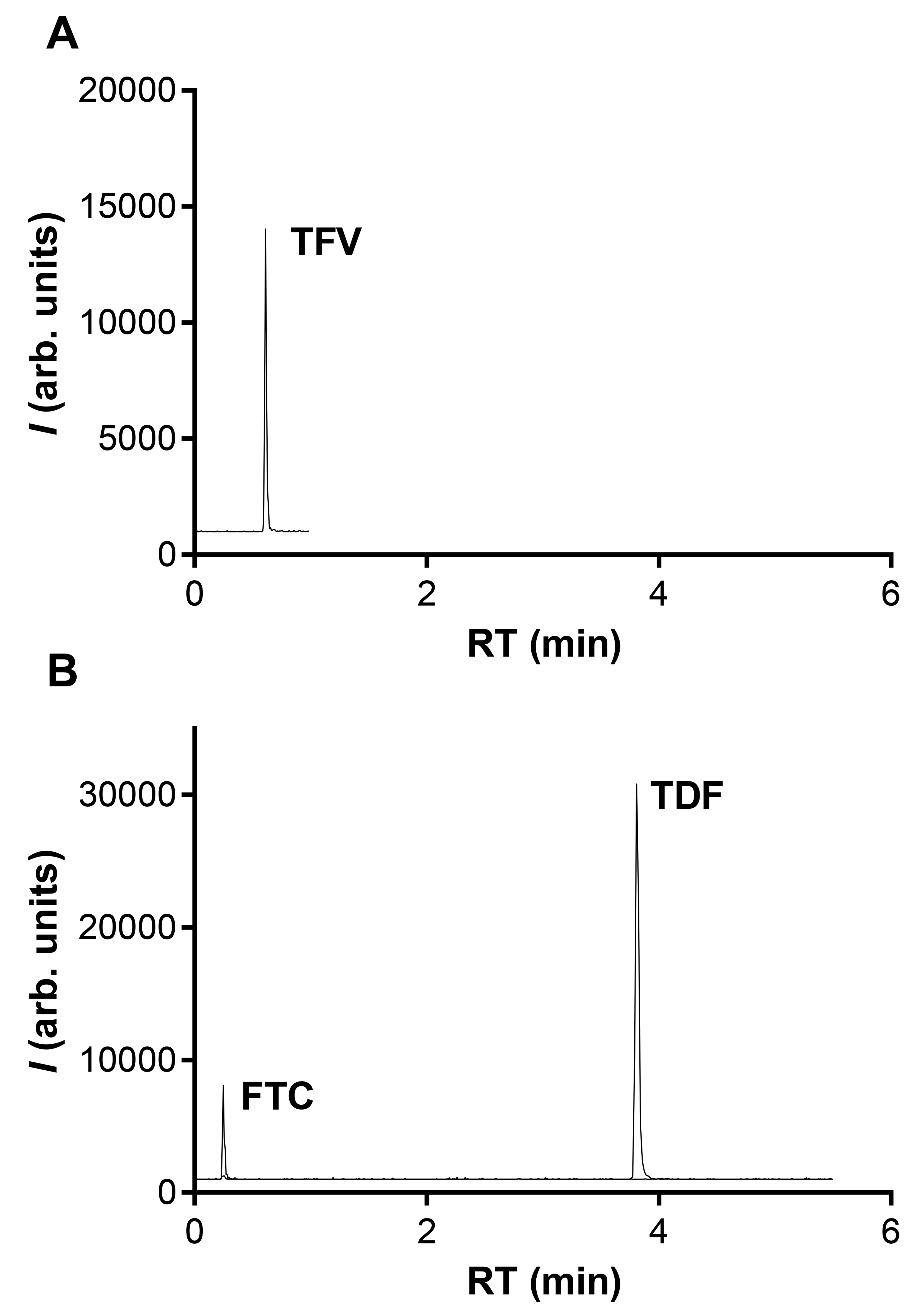

Supplement: S1 Fig — Sample LC-MS/MS chromatogram overlays (A) TFV (m/z, 288.1 → 176.2) and (B) TDF (m/z, 520.0 → 270.0), FTC (m/z, 248.0 → 130.0)—from a vaginal fluid sample collected during the TDF-FTC pod-IVR efficacy trial. The chromatograms were offset to illustrate the quality of the baseline. (TIF) [file pone.0157061.s001.tif]
