# Supplementary material for: Topical Delivery of Tenofovir Disoproxil Fumarate and Emtricitabine from Pod-Intravaginal Rings Protects Macaques from Multiple SHIV Exposures
Source: PLoS One. 2016 Jun 8;11(6):e0157061. doi: 10.1371/journal.pone.0157061 (PMC4898685; doi:10.1371/journal.pone.0157061)
Supplement: S1 Table — (DOCX) [file pone.0157061.s002.docx]

**S1 Table: Seroconversion (EIA and western blot) of TDF/FTC and control macaques during the challenge phase**

|  | Week of virus exposure | | | | | | | | | |
| --- | --- | --- | --- | --- | --- | --- | --- | --- | --- | --- |
| Animal | 1 | 2 | 4 | 6 | 8 | 10 | 12 | 14 | 16 | 18 |
| T^a^ 1 | **-**^c^ | **-** | **-** | **-** | **-** | **-** | **-** | **-** | **-** | **-** |
| T 2 | **-** | **-** | **-** | **-** | **-** | **-** | **-** | **-** | **-** | **-** |
| T 3 | **-** | **-** | **-** | **-** | **-** | **-** | **-** | **-** | **-** | **-** |
| T 4 | **-** | **-** | **-** | **-** | **-** | **-** | **-** | **-** | **-** | **-** |
| T 5 | **-** | **-** | **-** | **-** | **-** | **-** | **-** | **-** | **-** | **-** |
| T 6 | **-** | **-** | **-** | **-** | **-** | **-** | **-** | **-** | **-** | **-** |
| C^b^ 1 | **-** | **-** | **-** | **-** | **-** | **-** | **-** | **+** | **+** | **+** |
| C 2 | **-** | **-** | **-** | **-** | **+** | **+** | **+** | **+** | **+** | **+** |
| C 3 | **-** | **-** | **+**^d^ | **+** | **+** | **+** | **+** | **+** | **+** | **+** |

^a^ T-TDF/FTC macaques

^b^ C-Control macaques

^c^ (-) negative with EIA and western Blot

^d^ (+) positive by EIA and western blot
